# Effects of formulation on the bioavailability of lutein and zeaxanthin: a randomized, double-blind, cross-over, comparative, single-dose study in healthy subjects

**DOI:** 10.1007/s00394-012-0447-9

**Published:** 2012-09-30

**Authors:** Malkanthi Evans, Mareike Beck, James Elliott, Stephane Etheve, Richard Roberts, Wolfgang Schalch

**Affiliations:** 1 KGK Synergize, London, ON Canada; 2 DSM Nutritional Products Ltd., Kaiseraugst, Switzerland; 3 DSM Nutritional Products Inc., Parsippany, NJ USA; 4 Kemin Health, Des Moines, IA USA

**Keywords:** Xanthophylls, Carotenoids, Lutein, Zeaxanthin, *all*-*E*-lutein, Bioavailability

## Abstract

**Purpose:**

Lutein and zeaxanthin are macular pigments with a protective function in the retina. These xanthophylls must be obtained from the diet or added to foods or supplements via easy-to-use, stable formulations. The technique employed to produce these formulations may affect the bioavailability of the xanthophylls.

**Methods:**

Forty-eight healthy volunteers were randomized into this double-blind, cross-over study investigating the plasma kinetics of lutein provided as two different beadlet formulations. Subjects (*n* = 48) received a single dose of 20 mg of lutein as either a starch-matrix (“SMB”, FloraGLO^®^ Lutein 5 %) or as a cross-linked alginate-matrix beadlet (“AMB”, Lyc-O-Lutein 20 %) formulation. Plasma concentrations of lutein and zeaxanthin were measured at 0, 1, 3, 6, 9, 12, 14, 24, 26, 28, 32, 36, 48, 72, 168, and 672 h.

**Results:**

The mean plasma AUC_(0–72h)_, AUC_(0–672h)_, and *C*_max_ for total lutein and zeaxanthin and their *all*-*E*-isomers were significantly increased (*p* < 0.001) from pre-dose concentrations in response to SMB and AMB. There was no difference in lutein *T*_max_ between the two test articles. However, by 14 h post-dose, total plasma lutein increased by 7 % with AMB and by 126 % with SMB. Total lutein AUC_(0–72h)_ and AUC_(0–672h)_ were 1.8-fold and 1.3-fold higher, respectively, for SMB compared to AMB. Both formulations were well tolerated by subjects in this study.

**Conclusion:**

These findings confirm that the bioavailability of lutein and zeaxanthin critically depends on the formulation used and document a superiority of the starch-based over the alginate-based product in this study.

## Introduction

Lutein and zeaxanthin are xanthophyllic carotenoids found in fruits and vegetables and have been described as natural antioxidants [1, 2]. Humans are not capable of synthesizing carotenoids, and thus, their presence in human tissues is entirely of dietary origin [3]. On average, the combined daily dietary intake of lutein plus zeaxanthin ranges around 2 mg for the US population [4] but for some populations, such as South Pacific islanders, it may be as high as 26 mg per day due to their unusually high intake of fruits and vegetables high in these carotenoids [5]. Lutein is found in a number of human tissues including serum (0.1–1.23 μM), liver (0.1–3.0 μM), kidney (0.037–2.1 μM), and lung (0.1–2.3 μM) [6]. By far the highest concentration of these carotenoids (0.1–1 mM) is found in the human retina [7] providing evidence for active uptake or storage [8]. The macular region of the retina is yellow as a result of the presence of lutein and zeaxanthin [3]. Their specific location and physiochemical properties including their ability to absorb high-energy blue light and their capability to quench reactive oxygen species suggest that these carotenoids serve a protective function in the retina [9]. Previous studies showed that poor dietary intake or low plasma lutein and zeaxanthin concentrations are associated with low macular pigment density and an increased risk potential for age-related macular degeneration (AMD), an irreversible ocular condition that is the major cause of blindness in the elderly [10].

Although individual responses are known to differ markedly, many other factors could also play important roles during intestinal absorption, metabolism, and serum clearance of carotenoids, including interaction with other carotenoids [11]. Due to the solubility characteristics of carotenoids, the amount of fat consumed in conjunction with carotenoids appears to be an important factor in determining their bioavailability [12, 13].

In one of the most rigorous studies conducted previously, Thürmann et al. [14] showed that supplementation with 4.1 and 20.5 mg unesterified lutein increased plasma lutein concentrations approximately 3.5- and 10-fold, respectively. On the basis of previous studies, it may be hypothesized that dietary lutein and zeaxanthin in the form of lutein-containing supplements may increase the amount of serum lutein significantly upon the ingestion of lutein capsules [15]. As lutein in its crystalline form is unacceptable for use in tableted products for a variety of reasons, it is important to encapsulate the lutein into a powdered form. However, not all encapsulation materials that could be used in creating a powdered form are equally acceptable. For instance, one of the most common materials, bovine-derived gelatin, is infrequently used because of reasons associated with bovine spongiform encephalitis. Additionally, manufacturers of vitamins/dietary supplements require encapsulated materials that can withstand a wide range of tableting pressures placing significant restrictions upon the materials that can be used in the encapsulation of lutein and zeaxanthin. These same restrictions are believed to play a critical role in the bioavailability of these xanthophylls since the encapsulation must release these molecules during the digestive process in order for these carotenoids to reach the bloodstream.

While several single-dose, comparative pharmacokinetic (PK) studies have been conducted in human subjects using lutein or lutein esters [11–13], only one multiple-dose PK study [14] has been published. No comparative PK studies using two different sources of unesterified lutein have been published so far. Unpublished data indicated that materials and processes used in the encapsulation of lutein may affect lutein bioavailability. The present study was designed to compare, in human subjects, the bioavailability of lutein and zeaxanthin when ingested in two different formulations. Data gathered from Thürmann et al. [14] and other sources were used to establish many of the parameters used to design this study, including (but not limited to) the use of a single dose, the duration of the follow-up after lutein/zeaxanthin dosage administration, and the number of subjects employed.

## Subjects

Forty-eight subjects (24 males and 24 females) were recruited into the study from an available clinic volunteer database. To be eligible for enrollment, subjects were required to be healthy as confirmed by screening laboratory results, medical history, and physical examination, be between 18 and 65 years of age, have a BMI >20 and <30 kg/m^2^, have screening plasma lutein concentrations between 0.12 and 0.49 μmol/L, and agree to maintain current dietary habits throughout the duration of the study. Subjects using cholesterol lowering medications, supplements containing lutein or beta-carotene, medications that could affect drug or dietary supplement metabolism and excretion of drugs or dietary supplements, other natural health products including vitamins and minerals, or having an allergy or sensitivity to study supplement ingredients were excluded.

This study was reviewed by the Natural Health Products Directorate (NHPD), Health Canada, Ottawa, Ontario, Canada, and the Institutional Review Board Services (IRBS), Aurora, Ontario, Canada, and was unconditionally approved by the NHPD, and IRBS on July 30, 2009, and August 18, 2009, respectively.

This study was conducted in accordance with the ethical principles of the Declaration of Helsinki and its subsequent amendments. Informed consent was obtained from each subject at the screening visit prior to any study-related activities.

## Study design

The study was a single-center, randomized, double-blind, cross-over, 672-h bioavailability clinical investigation conducted at KGK Synergize Inc., London, Ontario, Canada. The study consisted of a two-day baseline period followed by a single-dose bioavailability phase that was followed by a second single-dose bioavailability phase 28 days later. This 28-day separation of the two phases was considered appropriate, since after 25 days plasma lutein levels are expected to have dropped to approximately 3 % of peak concentrations based on a terminal elimination half-life of 5 days as reported by Thürmann et al. [14].

At screening, informed consent was obtained and a medical history and concomitant therapies were reviewed. Height, weight, heart rate, and blood pressure were measured and BMI was calculated. A physical examination was performed and peripheral blood collected to determine complete blood count (CBC), electrolytes, glucose, creatinine, aspartate aminotransferase (AST), alanine aminotransferase (ALT), gamma glutamyltransferase (GGT), bilirubin, and lutein. Eligible subjects returned to the clinic for their baseline visits. At baseline (day −2 and day −1), concomitant therapies were reviewed, and fasting (12 h) blood samples were collected for analyses of plasma lutein and zeaxanthin.

At the start of the bioavailability Phase I (day 0), a fasting (12 h) blood sample was collected for pre-dose lutein and zeaxanthin analysis. The subject was then given a single AMB or SMB capsule (Time = 0 h) with breakfast provided immediately afterward (AMB capsules contained 20.9 mg lutein (2.2 % coefficient of variation, CV) and 1.55 mg zeaxanthin (2.3 % CV) per dose and SMB capsules contained 20.4 mg lutein (1.5 % CV) and 1.75 mg zeaxanthin (1.5 % CV) per dose). This capsule was taken orally by test subjects in the morning and witnessed by the study coordinator thereby ensuring compliance. Thereafter, blood samples were collected at 1, 3, 6, 9, 12, 14, 24, 26, 28, 32, 36, 48, 72, 168, and 672 h post-dose. All subjects received lunch following the 6-h sample and dinner after the 12-h sample. Subjects remained in the clinic from pre-dose until the 14-h sample collection; returned to the clinic fasting on day 1 for 24, 26, 28, 32, and 36 h post-dose blood collections and remained in the clinic for that period of time. Breakfast was provided immediately after the 24-h blood draw, lunch following the 28-h sample and dinner immediately following the 32-h sampling. Light snacks were provided between dinner and 36 h post-dose sampling. The food consumed at each meal time including all snacks were measured and recorded. The meals provided to subjects in the clinic during the first 36 h of the bioavailability phases did not include foods considered to be high in xanthophyll content. Subjects were permitted to leave the clinic after the 36 h post-dose blood sample. Subjects returned fasting (12 h) to the clinic for the 48, 72, and 168 h post-dose collections. Adverse events and concomitant therapies were reviewed at every visit.

Subjects returned to the clinic fasting (12-h fast) on the 28th day for Phase II of the study. The Phase II pre-dose (Time = 0 h) blood sample was the same as the 672-h Phase I post-dose sample. During this visit, adverse events and concomitant therapies were again reviewed. The subjects received one capsule of the second test article and all blood sampling and procedures remained exactly the same as in Phase I. The six meals and the snacks provided during the initial 36 h of the second bioavailability phase were of the same composition and amount as the meals provided during the first bioavailability phase. Subjects were not provided with caffeinated beverages during the first 36 h of either bioavailability phase.

## Sample size

A sample size calculation was performed based on the plasma lutein area under the curve [14]. Assuming a type I error rate (two-sided) of 0.05, an estimated standard deviation of 15.0, a correlation of 0.10 between the two study phases, and a 15 % loss to follow-up, 48 subjects were required to detect a between-formulation difference of 9.0 μmol h/L in the area under the curve for plasma lutein [16].

## Randomization

Enrolled subjects were stratified by gender to balance the two dosing sequences thus ensuring that an equal number of males and females were assigned to each sequence group (12 males and 12 females per dosing sequence). Subjects were randomized to one of two treatment sequences (AMB to SMB or SMB to AMB) in blocks of two. The Investigator was provided with two randomization schedules, one for males and one for females.

## Blinding

The test articles were labeled with the randomization number and order of treatment, that is, first dose and second dose, thereby blinding the identity of the test articles to the subject, the Investigator and all clinical site personnel directly involved in this study. The Investigator received sealed code envelopes for each subject enrolled into the study identifying which dosing sequence the subject received. A broken code required the patient to be withdrawn from the study. No premature unblinding occurred during the study.

## Lutein and zeaxanthin analysis

Plasma lutein and zeaxanthin measurements were performed at DSM’s Analytical Research Center (Kaiseraugst, Switzerland). Their concentrations were determined by normal-phase high-performance liquid chromatography, using published procedures [17]. Plasma samples were analyzed for zeaxanthin (*all*-*E* and total (=sum of *all*-*E* and Z-isomers)) and lutein (*all*-*E* and total (=sum of *all*-*E* and Z-isomers)). The xanthophylls were extracted from plasma (100 μL) with a 20 % mixture of *n*-hexane and chloroform (1,100 μL) after dilution with water (100 μL) and protein precipitation with ethanol (200 μL). After centrifugation, an aliquot (800 μL) of the clear supernatant fluid was dried under nitrogen at room temperature. The dried residue was quantitatively redissolved in the mobile phase (200 mL *n*-hexane and acetone; 19 %, by volume). The resulting solution was injected (100 μL) into a normal-phase HPLC system (Jasco, Japan) equipped with an autosampler (15 °C), a column oven (40 °C), an HPLC pump, and an ultraviolet–visible detector. Data acquisition, integration, and quantification were performed with Atlas Software (Thermo Labsystems). Quantification was performed by applying external calibration, without using internal standards. The separation was done on a polar column (Lichrosorb, Si60, 5 mm, 250 × 4 mm; Stagroma, Switzerland) with a mixture of *n*-hexane and acetone (19 %, by volume) at a flow rate of 1 mL/min. Xanthophylls were detected at a wavelength of 452 nm. The identification of the compounds was carried out by comparing the retention times with those of authentic reference standards of lutein and zeaxanthin (DSM Nutritional Products, Switzerland). To assess the daily and long-term laboratory performance of the HPLC plasma analytics, dedicated control plasma was used. The control plasma samples were analyzed at least 4 times/day during the study as described in [17]. In addition, the method was regularly checked for accuracy and precision (±15 %) by participation in inter-laboratory studies organized by the National Institute of Standard and Technologies (NIST, Gaithersburg, Maryland, US). The limit of detection (LOD) for lutein, zeaxanthin and their isomers was 0.002 μmol/L, and the lower limit of quantification (LLQ) for lutein, zeaxanthin and their isomers was 0.007 μmol/L.

## Test articles

The two test articles investigated in this study were Lyc-O-Lutein 20 % VBAF, an alginate-matrix beadlet formulation (“AMB”, LYCORED, Beer Sheva, Israel), and FloraGLO^®^ Lutein 5 % CWS-S/TG, a starch-matrix beadlet formulation (“SMB”, DSM Nutritional Products, Kaiseraugst, Switzerland). The test articles were filled into gelatin capsules opacified with titanium oxide and colored with red iron oxide by Temmler Werke, Munich, Germany. The AMB lutein beadlet material contained 203 mg/g of lutein and 15.3 mg/g of zeaxanthin. The respective capsules were accordingly filled with 98.5 mg of beadlets to contain exactly 20.9 mg of lutein and 1.55 mg zeaxanthin per capsule. This test article contained unesterified lutein in a cross-linked alginate-based formulation. The SMB lutein beadlet material contained 51 mg/g of lutein and 4.35 mg/g of zeaxanthin. The respective capsules were accordingly filled with 392.2 mg of these beadlets to contain exactly 20.4 mg of lutein and 1.75 mg zeaxanthin per capsule. This test article contained unesterified lutein in a starch-based matrix. Both test articles were assayed for lutein content and content uniformity by HPLC before initiation of the study and found to be acceptable.

## Statistical methods

The primary study endpoint was the 72-hour area under the curve for plasma lutein (AUC_0-72h_). Secondary endpoints included the maximum concentration (*C*_max_), the time at which the maximum concentration was observed for plasma lutein (*T*_max_) and AUC extended to include 672-h plasma concentrations, and finally the 72- and 672-h pharmacokinetic parameters (AUC_0-72h_, AUC_0–672h_, *C*_max_, and *T*_max_) for zeaxanthin. The area under the curve was calculated using the linear trapezoidal rule. In order to meet the assumption of normality, statistics on AUC and *C*_max_ were based on log transformed values for individual subjects [18]. One subject withdrew prior to the 672-h blood collection in their first dosing period (SMB). This subjects’ 672-h values were imputed using the subjects pre-dose values (time = 0 h) for the missing data point. The pre-dose values were used as it was expected that the plasma lutein and zeaxanthin concentrations would reach pre-dose levels after 672 h. This subject was included in the analysis of the 672-h bioavailability for SMB.

Descriptive statistics (mean and standard deviation) are reported for both test articles. Repeated measures analysis of variance was used to compare the two test articles. Subjects withdrawn prior to the second dosing period were excluded from the repeated measures analysis of variance. Tests for carry-over (between-sequence) and period effects were conducted [18]. Where values were reported as less than the lower limit of detection (LOD) or the lower limit of quantification (LLQ), a random value between 0 and the LLQ or LOD was assigned using SAS for the particular analyte being assessed. Adverse events which occurred within the study period were reported in detail, and the percentage of subjects experiencing adverse events were compared between the test articles using the Mainland–Gart test [18]. Probability values less than 0.05 denote statistically significant differences between test articles. SAS version 9.1 (Cary, NC, USA) was used to perform the statistical analysis.

## Results

**Fig. 1:**
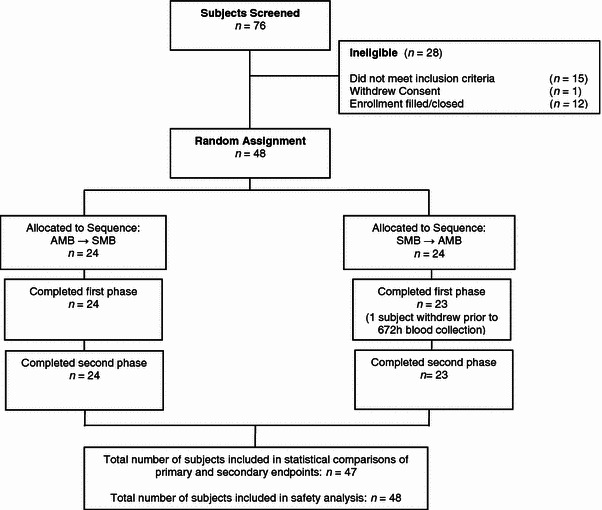
Diagram of study participant eligibility, enrollment, randomization, and follow-up

A total of 48 subjects (24 males and 24 females) were randomized to participate in the study. One subject withdrew due to personal reasons after the 168-h time point of the first dosing period and thus did not participate in the second dosing period (Fig. 1). Subjects presented with a mean age of 38.5 ± 14.0 years and mean BMI of 25.6 ± 3.3 kg/m^2^ (Table 1). After the initial washout period prior to the first treatment (day −1), subjects had a total lutein and total zeaxanthin plasma concentration of 0.198 ± 0.086 μmol/L and 0.067 ± 0.036 μmol/L, respectively, representing a lutein to zeaxanthin ratio of 3:1. No carry-over or period effects were observed for any of the results obtained from this study.

**Table 1:** Demographics and characteristics of all randomized subjects at baseline

	All subjects (*n* = 48)
Age (years)^a^	38.5 ± 14.0
BMI (kg/m^2^)^a^	25.6 ± 3.3
Mean systolic BP (mm Hg)^a^	112.1 ± 10.7
Mean diastolic BP (mmHg)^a^	70.1 ± 7.7
Mean heart rate (bpm)^a^	72.5 ± 7.7
Lutein^a^	
Total lutein (μmol/L)	0.198 ± 0.086
*all*-*E*-lutein (μmol/L)	0.166 ± 0.071
Zeaxanthin^a^	
Total zeaxanthin (μmol/L)	0.067 ± 0.036
*all*-*E*-zeaxanthin (μmol/L)	0.052 ± 0.026
Gender—female^b^	24 (50.0 %)
Race/ethnicity^b^	
Asian-oriental	2 (4.2 %)
Black	2 (4.2 %)
Caucasian	42 (87.5 %)
East Indian	2 (4.2 %)
Alcohol use^b^	
Daily/weekly	13 (27.1 %)
None	7 (14.6 %)
Occasional	28 (58.3 %)
Tobacco use^b^	
Current	7 (14.6 %)
Former	14 (29.2 %)
Never	27 (56.3 %)

^a^Continuous variables are displayed as mean with standard deviation

^b^Categorical variables are displayed as *n* (%)

### Lutein bioavailability

The mean plasma total lutein and *all*-*E*-lutein *C*_max,_ AUC_(0–72h)_ and AUC_(0–672h)_ were significantly higher (*p* < 0.001) compared to baseline for each of the two test products. However, the response to SMB was also significantly different from AMB with SMB being more bioavailable (Tables 2, 3). Though the time to reach maximum concentration (*T*_max_) was not significantly different for total lutein or *all*-*E*-lutein between the two test articles during the first 72 h (Table 2), *T*_max_ occurred sooner in response to SMB for total lutein and *all*-*E*-lutein as compared to AMB (17.6 vs. 20.6 h and 17.4 vs. 19.8 h, respectively).

**Table 2:** Lutein and zeaxanthin bioavailability measured by the response of plasma concentrations over 72 h after a single dose of AMB or SMB

	AMB (*n* = 47)	*p* value^a^	SMB (*n* = 48)	*p* value^a^	*p* value^b^
Total lutein					
Dose (mg)	20.9		20.4		
*C*_max_ (μmol/L)^c^	0.238 ± 0.091	<0.001	0.460 ± 0.169	<0.001	<0.001
*C*_max_ (increase from *t* = 0 h) (μmol/L)	0.055 ± 0.054	<0.001	0.279 ± 0.130	<0.001	<0.001
*T*_max_ (h)	20.6 ± 22.5	<0.001	17.6 ± 7.8	<0.001	0.432
*C*_14h_ (increase from *t* = 0 h) (μmol/L)	0.013 ± 0.046	0.059	0.228 ± 0.154	<0.001	<0.001
AUC_(0–72h)_ (μmol h/L)^c^	13.032 ± 4.847	<0.001	23.508 ± 8.539	<0.001	<0.001
*all*-*E*-lutein					
*C*_max_ (μmol/L)^c^	0.200 ± 0.077	<0.001	0.416 ± 0.157	<0.001	<0.001
*C*_max_ (increase from *t* = 0 h) (μmol/L)	0.046 ± 0.047	<0.001	0.264 ± 0.124	<0.001	<0.001
*T*_max_ (h)	19.8 ± 21.1	<0.001	17.4 ± 7.5	<0.001	0.498
*C*_14h_ (increase from *t* = 0 h) (μmol/L)	0.011 ± 0.039	0.056	0.219 ± 0.143	<0.001	<0.001
AUC_(0–72h)_ (μmol h/L)^c^	10.965 ± 4.040	<0.001	20.801 ± 7.874	<0.001	<0.001
Total zeaxanthin					
Dose (mg)	1.55		1.75		
*C*_max_ (μmol/L)^c, d^	0.077 ± 0.032	<0.001	0.083 ± 0.038	<0.001	0.164
*C*_max_ (increase from *t* = 0 h)(μmol/L)^d^	0.018 ± 0.017	<0.001	0.028 ± 0.021	<0.001	0.009
*T*_max_ (h)	17.5 ± 20.9	<0.001	19.4 ± 12.1	<0.001	0.595
*C*_14h_ (increase from *t* = 0 h) (μmol/L)^d^	0.003 ± 0.014	0.225	0.008 ± 0.026	0.046	0.315
AUC_(0–72h)_ (μmol h/L)^c, d^	4.110 ± 1.785	<0.001	4.414 ± 2.321	<0.001	0.175
*all*-*E*-zeaxanthin					
*C*_max_ (μmol/L)^c, d^	0.060 ± 0.024	<0.001	0.067 ± 0.031	<0.001	0.032
*C*_max_ (increase from *t* = 0 h) (μmol/L)^d^	0.013 ± 0.012	<0.001	0.023 ± 0.015	<0.001	<0.001
*T*_max_ (h)	19.4 ± 21.0	<0.001	20.4 ± 12.1	<0.001	0.757
*C*_14h_ (increase from *t* = 0 h) (μmol/L)^d^	0.003 ± 0.010	0.057	0.008 ± 0.019	0.006	0.135
AUC_(0–72h)_ (μmol h/L)^c, d^	3.253 ± 1.337	<0.001	3.569 ± 1.903	<0.001	0.082

All values are expressed as mean with standard deviation

^a^Within group comparisons for the difference from zero were made using *t* tests. Probability values *p* < 0.05 are statistically significant

^b^Between group comparisons were made using analysis of variance (ANOVA). Probability values *p* < 0.05 are statistically significant

^c^Data were log transformed prior to statistical comparisons

^d^Zeaxanthin values for SMB were adjusted (multiplied by the factor (1.55/1.75)) to correct for the difference in dose of zeaxanthin between the two study products

**Table 3:** Lutein and zeaxanthin bioavailability measured by AUC (μmol h/L) in plasma over 672 h after a single dose of AMB or SMB

	AMB (*n* = 47)	*p* value^a^	SMB (*n* = 48^b^)	*p* value^a^	*p* value^c^
Total lutein	120.8 ± 47.4	<0.001	162.8 ± 70.2	<0.001	<0.001
*all*-*E*-lutein	102.7 ± 41.1	<0.001	139.1 ± 63.3	<0.001	<0.001
Total zeaxanthin^d^	40.3 ± 22.3	<0.001	38.4 ± 28.6	<0.001	0.459
*all*-*E*-zeaxanthin^d^	32.4 ± 17.5	<0.001	30.9 ± 24.4	<0.001	0.396

All values are expressed as mean with standard deviation

^a^Within group comparisons for the difference from zero were made using *t* tests. Probability values *p* < 0.05 are statistically significant

^b^One subject withdrew prior to the 672 h blood collection in their first dosing period (SMB). This subjects’ 672 h values were imputed using the subjects’ pre-dose values (*t* = 0 h) for the missing data and the subject included in the analysis of the 672 h bioavailability for SMB

^c^Between group comparisons were made using analysis of variance (ANOVA). Probability values *p* < 0.05 are statistically significant

^d^Zeaxanthin values for SMB were adjusted (multiplied by the factor (1.55/1.75)) to correct for the difference in dose of zeaxanthin between the two study products

**Fig. 2:**
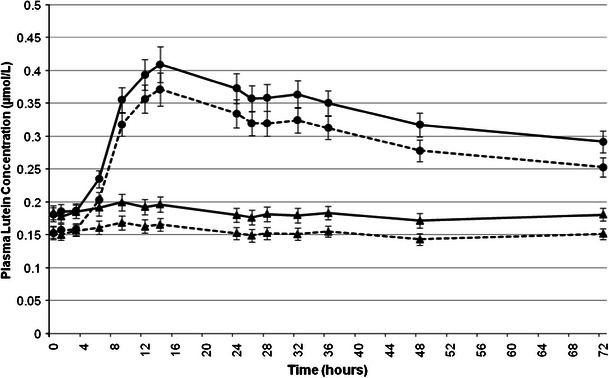
Mean plasma total lutein (*solid line*) and *all*-*E*-lutein (*dotted line*) concentrations pre-dose and over a 72-h period following administration of AMB (*triangle*) or SMB (*circle*) each containing, respectively, 20.9 or 20.4 mg of lutein and 1.55 or 1.75 mg of zeaxanthin. Data are expressed as mean ± SEM

**Fig. 3:**
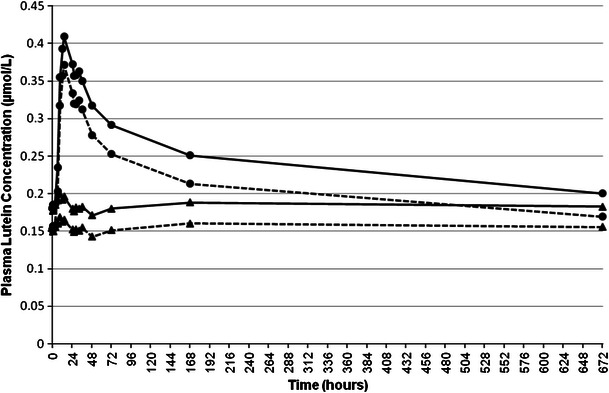
Mean plasma total lutein (*solid line*) and *all*-*E*-lutein (*dotted line*) concentrations pre-dose and over a 672-h period following administration of AMB (*triangle*) or SMB (*circle*) each containing, respectively, 20.9 or 20.4 mg of lutein and 1.55 or 1.75 mg of zeaxanthin. Data are expressed as mean ± SEM

The mean plasma profile of *all*-*E*-lutein (0–72 h and 0–672 h) followed a comparable pattern to the mean plasma profile of total lutein for both test articles (Figs. 2, 3). The mean increase in plasma for total lutein and *all*-*E*-lutein concentrations from 0 (pre-dose) to 14 h after administration of AMB was 0.013 μmol/L (7.1 %) and 0.011 μmol/L (7.1 %), respectively. The mean increase in plasma total lutein and *all*-*E*-lutein concentrations from 0 (pre-dose) to 14 h after administration of SMB was 0.228 μmol/L (126.0 %) and 0.219 μmol/L (144.1 %), respectively.

At 72 h post-administration (Fig. 2) of SMB, *all*-*E*-lutein remained higher than pre-dose concentrations (∆ = 0.100 μmol/L or 65.4 %). However, when subjects were on AMB, mean plasma *all*-*E*-lutein concentrations reached pre-dose concentrations (∆ = −0.003 μmol/L or −1.9 %) by 72 h. Total plasma lutein showed similar results.

By 672 h (Fig. 3), plasma *all*-*E*-lutein concentrations remained higher than pre-dose (∆ = 0.017 μmol/L or 11.1 %) for SMB and remained similar to pre-dose concentrations (∆ = 0.002 μmol/L or 1.3 %) for AMB. Changes in plasma total lutein concentrations followed a similar pattern to plasma *all*-*E*-lutein at 672 h.

### Zeaxanthin bioavailability

Mean increases from baseline in plasma total zeaxanthin and *all*-*E*-zeaxanthin *C*_max_ were significantly higher (*p* < 0.01) in response to SMB as compared to AMB (Table 2). The difference in zeaxanthin dose between AMB (1.55 mg) and SMB (1.75 mg) was accounted for in the calculations for Tables 2 and 3. T_max_ was not statistically significant between groups. Plasma total zeaxanthin and *all*-*E*-zeaxanthin AUC_(0-72h)_ and AUC_(0-672h)_ were not different between treatments.

**Fig. 4:**
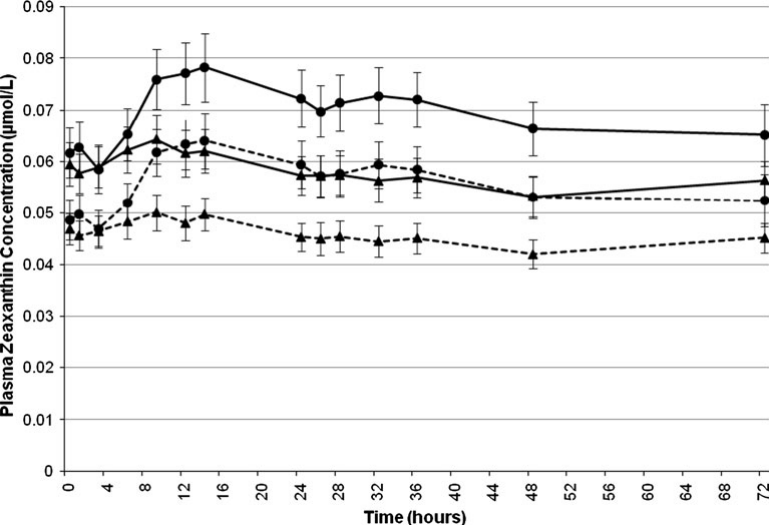
Mean plasma total zeaxanthin (*solid line*) and *all*-*E*-zeaxanthin (*dotted line*) concentrations pre-dose and over a 72-h period following administration of AMB (*triangle*) or SMB (*circle*) each containing, respectively, 20.9 or 20.4 mg of lutein and 1.55 or 1.75 mg of zeaxanthin. Data are expressed as mean ± SEM

**Fig. 5:**
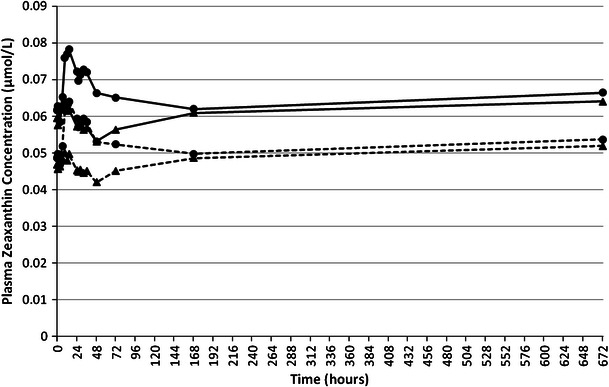
Mean plasma total zeaxanthin (*solid line*) and *all*-*E*-zeaxanthin (*dotted line*) concentrations pre-dose and over a 672-h period following administration of AMB (*triangle*) or SMB (*circle*) each containing, respectively, 20.9 or 20.4 mg of lutein and 1.55 or 1.75 mg of zeaxanthin. Data are expressed as mean ± SEM

The mean plasma profile of *all*-*E*-zeaxanthin (0–72 h and 0–672 h) followed a comparable pattern to the mean plasma profile of total zeaxanthin for both test articles (Figs. 4, 5). The mean increase in plasma total zeaxanthin and *all*-*E*-zeaxanthin concentrations from 0 (pre-dose) to 14 h after administration of AMB was 0.003 μmol/L (5.1 %) and 0.003 μmol/L (6.4 %), respectively. The mean increase in plasma total zeaxanthin and *all*-*E*- zeaxanthin concentrations from 0 (pre-dose) to 14 h after administration of SMB was 0.008 μmol/L (12.9 %) and 0.008 μmol/L (16.3 %), respectively (Table 2).

Seventy-two hours post-administration (Figs. 4, 5) of AMB, mean plasma total zeaxanthin and *all*-*E*-zeaxanthin concentrations were decreased to lower levels than those seen pre-dose (∆ = −0.003 μmol/L or −5.1 % and ∆ = −0.002 μmol/L or −4.3 %, respectively). For SMB, though values had reduced from peak plasma concentrations, total plasma zeaxanthin concentrations remained higher than pre-dose concentrations (∆ = 0.003 μmol/L or 4.8 %) and plasma *all*-*E*-zeaxanthin concentrations remained higher than pre-dose concentrations (∆ = 0.003 μmol/L or 6.1 %) 72 h post-administration (Fig. 4).

It is noteworthy that mean plasma total zeaxanthin and mean plasma *all*-*E*-zeaxanthin concentrations were below pre-dose concentrations at 24, 26, 28, 32, 36, 48, and 72 h post-administration of AMB. This pattern was not seen for SMB, where values remained above pre-dose concentrations during the same period. The mean drop below pre-dose concentrations reported for AMB from the 24 through 72 h blood collections ranged between −0.002 and −0.006 μmol/L (−3.4 to −10.2 %) for total zeaxanthin and between −0.002 and −0.005 μmol/L (−4.2 to −10.6 %) for *all*-*E*-zeaxanthin.

### Adverse events

A total of 27 adverse events were reported during the study (13 for AMB and 14 for SMB). Only one of these adverse events was categorized by the Investigator as being related to the test article, specifically to SMB. This event, a loose bowel movement, occurred on the day the test article was administered and resolved the following day without the need for concomitant medication.

## Discussion

Previous studies have been conducted using multiple dosing regimens and variable doses to determine the plateau concentration of lutein in the bloodstream. The current study was designed to assess the effect of different formulation technologies on the bioavailability profile of lutein and zeaxanthin after single oral doses of two comparative test articles both of which contained lutein and zeaxanthin, specifically in a starch-based or in an alginate-based matrix.

SMB demonstrated greater bioavailability than AMB exhibiting a 126.0 % increase at 14 h in total lutein and a 144.1 % increase from pre-dose in its principle isomer *all*-*E*-lutein while AMB showed an 7.1 % increase in total lutein and a 7.1 % increase from pre-dose in *all*-*E*-lutein. Seventy-two hours post-administration, the plasma concentrations of total lutein and *all*-*E*-lutein remained approximately 65 % higher than pre-dose values for SMB, whereas plasma values were at or below pre-dose concentrations for AMB by 72 h.

Although to a much lower degree due to the lower dose, zeaxanthin plasma profiles were similar to those of lutein, SMB performed better than AMB with a 12.9 % increase at 14 h from pre-dose in total zeaxanthin and a 16.3 % increase in its principle isomer *all*-*E*-zeaxanthin, while AMB showed an 5.1 % increase at 14 h from pre-dose in total zeaxanthin and a 6.4 % increase in *all*-*E*-zeaxanthin. Bioavailability of total and *all*-*E*-zeaxanthin in response to AMB exhibited an absorption pattern limited to the first 24 h post-test article administration followed by a decrease in blood concentrations below pre-dose levels. There was an increase in plasma concentrations after 72 h continuing through 672 h, perhaps suggesting a dietary influence. However, SMB demonstrated a plasma zeaxanthin profile that was maintained for greater than 72 h post-supplementation and, similar to AMB, the profile showed an increase in plasma values from 168 to 672 h. The serum profiles of *all*-*E*-lutein and *all*-*E*-zeaxanthin were similar to and closely followed the profiles of total lutein and total zeaxanthin suggesting that *all*-*E*-lutein and *all*-*E*-zeaxanthin are the predominant isomers in the plasma. The profile for the 72 h total lutein mimicked that of total zeaxanthin during the initial 72 h in response to SMB with an initial peak seen after 14 h and a second peak of lesser magnitude around 32 h.

It is interesting that this second peak appears in the profiles of both zeaxanthin (Fig. 4) as well as lutein (Fig. 2) and reaches maximum values after approximately 32 h in each case. The appearance of this second peak was not observed by Yao et al. [19] who measured lutein in the bloodstream of humans using a ^13^C tracer technique. However, the latter study included only one measurement of plasma lutein in the interval between 16 and 48 h, namely at 24 h. The lack of additional measurements within this timeframe when the second peak was observed in the present study, probably accounts for the differences observed. This second peak, visible for SMB only, could be explained by the general characteristics of carotenoid absorption. After ingestion of a single dose of β-carotene, a similar second plasma concentration peak has been reported [20]. The authors have concluded that the early rise in circulating β-carotene concentrations is caused by the intestinal input, whereas hepatic secretion is the source of later increases. It is likely that the xanthophylls behave similar to beta-carotene. Additionally, the second peak may arise from further release of xanthophylls into the circulation via newly synthesized chylomicrons from the intestine induced by a subsequent meal (fat). Such distinctive profiles in plasma response were not seen with AMB.

Statistical analysis of pharmacokinetic parameters demonstrated that total and *all*-*E*-lutein were significantly increased in the plasma in response to SMB. Mean total lutein and *all*-*E*-lutein AUC_(0–72h)_ were significantly increased (*p* < 0.001) in response to SMB as compared to AMB. Mean maximum plasma total lutein and *all*-*E*-lutein concentrations (*C*_max_) were significantly (*p* < 0.001) higher in subjects after administration of SMB. Though the time to reach maximum concentration (*T*_max_) was not significantly different between test products as measured by total or *all*-*E*-lutein, there was a faster response to SMB for total lutein and *all*-*E*-lutein as compared to AMB.

Numerous studies in the literature attest to the importance of the role of lutein and zeaxanthin in the prevention of age-related eye diseases in high-risk populations. In the course of the Lutein Antioxidant Supplementation Trial (LAST), a double-blind, placebo-controlled trial in 90 patients with atrophic AMD, 10 mg of lutein was supplemented for 1 year. Along with increases in macular pigment optical density, there was net improvement in several visual function parameters (glare and contrast sensitivity, visual acuity) in addition to a reversal of the symptoms of AMD indicating a potentially preventative activity against the development of AMD [21]. Nutritional studies correlating the effects of high dietary intake of antioxidants with protection against AMD reported that higher intakes of carotenoids were associated with a reduced risk of exudative neovascular macular degeneration [22]. The carotenoids lutein and zeaxanthin obtained principally from dark green, leafy vegetables such as spinach, kale, collard greens, mustard greens, and turnip greens were most strongly associated with reduced risk of AMD. Additionally, several prospective studies have reported that higher intakes of lutein and zeaxanthin were associated with decreased risk of cataracts [23]. After a 10-year follow-up, women consuming the most lutein and zeaxanthin had an 18 % lower risk of developing cataracts than those who consumed the least. More recently, older women with high dietary concentrations of lutein and zeaxanthin have been associated with decreased prevalence of nuclear cataracts [24].

Knowledge relating to the formulation of supplements and the pharmacokinetics of lutein absorption is critical to a better understanding of plasma bioavailability of these carotenoids. A variation of lutein from different food sources [25, 26] and the vast individual variation in macular accumulation and its variance in target populations [11] make it important that bioavailability studies research the pharmacokinetics of supplements prior to the implementation of long-term clinical trials. Furthermore, due to the fact that the polarities of lutein and zeaxanthin are similar, most researchers report combined values for lutein and zeaxanthin when reporting results. In the current study, plasma samples were analyzed for the xanthophylls lutein and zeaxanthin and their *all*-*E*-isomers thereby providing a more comprehensive assessment of the availability of the prevalent isomer in the plasma. Thus, the data generated from this study provide clear kinetics of the two materials evaluated after a single dose and allowed for the assessment of the bioavailability of the materials. In light of the high prevalence of eye disease in aging populations and the impact of lutein and zeaxanthin in its prevention, the results of the current study are significant.

Of the subjects enrolled into the current study, 87.5 % were White, while 4.2 % were Asian–Oriental, 4.2 % Black, and 4.2 % East Indian; 14.6 % of enrolled subjects were current smokers. Pooled data from several studies have identified that there is a strong age-related increase in AMD in people of European descent with significant increases in rates in both men and women older than 80 years of age [27].

In this population of subjects, a single dose of SMB resulted in a 126.0 % increase in total plasma lutein and a 144.1 % increase from pre-dose in its principle isomer *all*-*E*-lutein within the first 14 h as well as a significant increase in AUC_(0–72h)_ for total plasma lutein, *all*-*E*-lutein, total zeaxanthin and *all*-*E*-zeaxanthin and AUC_(0–672h)_ for total plasma lutein and *all*-*E*-lutein. AUC values were significantly higher than those reached after AMB administration, demonstrating the superiority of SMB over AMB. Additionally, the data gathered should be helpful to future research and clinical studies in relation to determining optimal dosing regimens and anticipated blood concentrations of lutein and zeaxanthin from the dosages chosen.

Although case–control studies suggest a combined dose of 6 mg of lutein and zeaxanthin per day for reducing the risk of AMD, the average North American ingests only 1–2 mg of lutein daily from their diet [22, 28]. This may lead to a deficit of these important carotenoids. With the dramatic increase in age-related eye diseases, it becomes very important to more thoroughly understand the issues associated with the bioavailability of lutein and zeaxanthin supplement formulations and their potential impact upon target populations.
